# *In Vitro* and *In Vivo* Determinations of The Anti-GDNF Family Receptor Alpha 1 Antibody in Mice by Immunochemistry and RT-PCR 

**DOI:** 10.22074/ijfs.2020.6051

**Published:** 2020-10-12

**Authors:** Hossein Azizi, Amirreza Niazi Tabar, Thomas Skutella, Mostafa Govahi

**Affiliations:** 1 Faculty of Biotechnology, Amol University of Special Modern Technologies, Amol, Iran; 2 Institute for Anatomy and Cell Biology, Medical Faculty, University of Heidelberg, Heidelberg, Germany

**Keywords:** Analysis, Embryonic Stem Cells, *GFRa1*, Pluripotent Stem Cells

## Abstract

**Background:**

The glial cell-derived neurotrophic factor (GDNF) family plays essential roles in the maintenance, growth, regulatory and signalling pathways of spermatogonial stem cells (SSCs). In this study, we analysed the ex- pression of anti-GDNF family receptor alpha 1 antibody (*GFRa1*) by immunohistochemistry (IHC), immunocyto- chemistry (ICC), Fluidigm real-time polymerase chain reaction (RT-PCR) and flow cytometry analyses.

**Materials and Methods:**

In this experiment study, ICC, IHC, Fluidigm RT-PCR and flow cytometry were used to analyse the expression of the germ cell marker *GFRa1* in testis tissue and SSC culture.

**Results:**

IHC analysis showed that there were two groups of *GFRa1* positive cells in the seminiferous tubules based on their location and expression shape - a small round punctuated shape on the basal compartment donut shape and a C-shaped expression located between the basal and the luminal compartments of the seminiferous tubules. OCT4 and PLZF positive cells may have similar patterns of expression as the first group. Assessment of the seminiferous tubule sections demonstrated that about 27% of the SSCs were positive for *GFRa1*. Fluidigm RT-PCR confirmed the significant expression (P<0.001) of *GFRa1* in the SSCs compared to testicular stromal cells (TSCs). Flow cytometry analysis demonstrated that about 75% of the isolated SSCs colonies were positive for *GFRa1*.

**Conclusion:**

The results indicated that *GFRa1* had a specific expression pattern both in vivo and in vitro. This finding could be helpful for understanding the proliferation, maintenance and signalling pathways of SSCs, and differentiation of meiotic and haploid germ cells.

## Introduction

In the mammalian testis, spermatogonial stem cells (SSCs) are located on the basal membrane of seminiferous tubules and are essential for normal spermatogenesis. SSCs can be established in adherent and nonadherent culture systems. The self-renewal and maintenance of SSCs during an in vitro culture depends on the presence of soluble growth factors and adhesion molecules. SSCs express different surface markers, including anti-GDNF family receptor alpha 1 antibody (GFRa1) (1, 2) α6 (CD49) and β1 (CD29) integrins (3, 4), CD9 (5), E-cadherin (6), and THY-1 (CD90) (7, 8). The GFRa1 receptor is expressed in undifferentiated spermatogonia cells in rodents and has been used as a marker for the isolation of undifferentiated SSCs (9). GFRa1 is a co-receptor that recognizes the glycosylphosphatidylinositol-linked glial cell-derived neurotrophic factor (GDNF) family of ligands. GDNF is a main growth factor for *in vitro* cultivation of SSCs and supports the survival of neuronal cells throughout the regulation of cyclic adenosine 3´, 5´-monophosphate (cAMP)-dependent signalling pathways (10, 11). In mammalian testes, GDNF affects the target cells by binding to a receptor complex that consists of receptor tyrosine kinase Ret (C-RET) and GFRa1 (12). During in vitro cultivation of testicular germ stem cells, the GDNF molecule regulates both self-renewal and proliferation of SSCs, prevents SSC differentiation and activates the in vivo maintenance of the stem cell pool (13-16). When two soluble growth factors, GFRa1 and fibroblast growth factor 2 (FGF2), are combined with GDNF, they enhance both proliferation and long-term expansion of cultivated germline stem cells (GSCs) (13). Similarly, GFRa1 combined with the growth fac tors FGF, LIF and GDNF supports the short-term cultivation of rat SSCs (17).

The aim of the present investigation was to understand the localization and pattern of *GFRa1* gene expression in the testis section and in generated SSCs and testicular stromal cells (TSCs). The results showed that GFRa1 expression was distributed above the base membrane of the testicular lumen, which suggested that GFRa1 plays a crucial role in the proliferation and self-renewal of germ stem cells in testes. The GFRa1 expression pattern would be valuable for innovative future researches in the fields of reproductive biology and biotechnology.

## Materials and Methods

### Digestion, characterization and culture of testicular cells

In this experimental study, the ethical committee of Amol University of Special Modern Technologies (IR.AUMST.REC.1398.03.07) approved the animal experiments. Testis cells from C57BL/6 mice (7-week-old) were placed in an enzymatic digestion solution that contained DNAse (0.5 mg/ml, Sigma Aldrich, USA), collagenase (0.5 mg/ml, Sigma Aldrich, USA) and dispase (0.5 mg/ml, Sigma Aldrich, USA) in an HBSS buffer (PAA, USA). After characterizing the SSCs, digested testicular cells were filtered through a cell strainer and were cultured in GSCs culture media at 37˚C and 5% CO_2_ in air. This media contained StemPro-34 medium, 1% L-glutamine (PAA, USA), 1% N2-supplement (Invitrogen, USA), 6 mg/ml D + glucose (Sigma Aldrich, USA), 1% penicillin/streptomycin (PAA, USA), 5 μg/ml bovine serum albumin (Sigma Aldrich, USA), 0.1% s-mercaptoethanol (Invitrogen, USA), 30 ng/ml oestradiol (Sigma Aldrich, USA), 60 ng/ml progesterone (Sigma Aldrich, USA), 1% non-essential amino acids (PAA, USA), 10 ng/ml FGF (Sigma Aldrich, USA), 100 U/ml human LIF (Millipore), 1% MEM vitamins (PAA, USA), 8 ng/ml GDNF (Sigma Aldrich, USA), 20 ng/ml epidermal growth factor (EGF, Sigma Aldrich, USA), 30 μg/ml pyruvic acid (Sigma Aldrich, USA), 1% ES cell qualified FBS, 100 μg/ml ascorbic acid (Sigma Aldrich, USA) and 1 μl/ml DL-lactic acid (Sigma Aldrich, USA).

### RNA extraction and real-time polymerase chain reaction analysis

Total RNA was extracted from the SSCs and TSCs with a NucleoSpin^®^ RNA II Kit (Macherey-Nagel, Duren, Germany) for real-time polymerase chain reaction (RT-PCR) analysis. In the next step, RNA samples were decontaminated with DNase I (EN0521, Fermentas, USA) to remove genomic DNA contamination. cDNA was synthesized with oligo (dT)18, total RNA (2 μg) and a RevertAidTM H Minus First Strand cDNA Synthesis Kit (K1622, Fermentas). The PCR reactions were carried out using a Mastercycler gradient machine (Eppendorf, Germany). The cDNA samples were exposed to PCR amplification by GFRa1 primers under the following reaction conditions: initial denaturation at 94˚C for 5 minutes, 30 cycles of denaturation at 94°C for 30 seconds, annealing temperature at 59-70°C for 45 seconds, extension time for 45 seconds at 72°C, and a final polymerization at 72°C for 10 minutes. The PCR products were observed using 1.6% agarose gel electrophoresis, stained with ethidium bromide solution (10 μg/ml), and then visualized and photographed with a UV transilluminator (UVIDOC, UK). The forward and reverse primer used for *GFRa-1* was as follow:

F: 5´-ACTCCTGGATTTGCTGATGTCGG-3´

R: 5´-CGCTGCGGCACTCATCCTT-3´ (product size: 193 bp) (18, 19).

### Gene expression analyses on the Fluidigm Biomark system

The expression level of the *GFRa1* Mm01253716_m1 gene in SSCs and TSCs was examined by the Fluidigm Biomark system. Glyceraldehyde-3-phosphate dehydrogenase (GAPDH) Mm99999915-g1 was the reference gene for normalization. SSCs and TSCs were picked up with a micromanipulator technique, lysed with a solution of lysis buffer that contained 9 μl RT-PreAmp Master Mix (5.0 μl Cells Direct 2× Reaction Mix, Invitrogen, USA), 2.5 μl 0.2× assay pool and 1.3 μl TE buffer, 0.2 μl RT/Taq Superscript III (Invitrogen, USA). Then, the amount of the amplified product of RNA-targeted copies was examined with TaqMan real-time PCR on a BioMark Real-Time Quantitative PCR (qPCR) system. Samples were analysed in two technical repeats. The Ct values were calculated using Excel and GenEx software (20-22).

### Immunocytochemical staining

Isolated SSCs from the testes were fixed with 4% paraformaldehyde, permeabilised with 0.1% Triton X-100/PBS, blocked with 1% BSA/PBS and incubated with primary antibody GFRa1 (Sigma Aldrich, USA). The process was followed by an overnight incubation (~16 hours) of fluorochrome species-specific secondary antibody at 4°C. The labelled cells were identified by simple nuclear counterstain with 0.2 μg/ml of 4′, 6-diamidino-2-phenylindole (DAPI) dye. The positive cells labelled with antibodies were visualised with a confocal laser scanning microscope Zeiss LSM 700 and images of the cells were obtained using a Zeiss LSM-TPMT camera (20, 23, 24).

### Tissue processing for immunohistochemical staining

Testicular tissue was picked up after decapsulation of tunica albuginea, washed with PBS and fixed in 4% paraformaldehyde. The tissue was dehydrated during tissue processing and surrounded in Paraplast Plus. In the next step, the tissue was cut with a microtome, usually with a thickness of around 8-10 μm. Sections from the testes tissues were mounted on Hydrophilic Plus slides and stored at room temperature until use. During the immunohistochemical staining process, the slides were washed by xylene and slowly dehydrated through a series of decreasing concentrations of ethanol. Before staining, antigen retrieval was done by the heat-induced epitope retrieval (HIER) method at 95°C for 20 minutes and the non-specific binding sites in the tissue sections were blocked with 10% serum and 0.3% Triton in PBS. Then, the tissue sections were incubated with primary antibody *GFRa1* (Sigma, USA) and species-specific secondary antibody. The labelled cells were characterised under a confocal laser scanning microscope Zeiss LSM (20).

### Flow cytometry analysis

After determining the cell viability by trypan blue staining, the cells were resuspended in PBS/FBS staining buffer and incubated with cell surface primary antibody to GFRa1 conjugated with fluorochrome (APC, R&D Systems) for one hour. The samples were washed and a flow cytometry analysis was performed with a BD FACSCalibur flow cytometer. The acquired results were analysed with BD CellQuest Pro software.

### Statistical analysis

Statistical analysis was performed using IBM SPSS Statistics for Windows, Version 25.0 (IBM Corp., Armonk, NY, USA). The comparison of GFRa1 expression in the SSCs and TSCs groups was analysed by the independent samples t test. P<0.05 was considered to be statistically significant.

## Results

The location of the germ cell marker GFRa1 in the seminiferous tubule of the mouse testis was analysed as the first study of this experiment. We observed two distinct populations of GFRa1 positive cells based on their location and pattern of expression in the seminiferous tubules of the mice. The first group of GFRa1 positive cells had a small round punctuated expression and were located at the epithelium of the seminiferous tubules. The number in the first group was much lower than the Second group. The second group of GFRa1 positive cells was situated between the basal and the luminal compartment of seminiferous tubules and had a donut and C-shaped expression. The OCT4 and PLZF positive cells and the first population of GFRa1 positive cells that were located in the basal part of seminiferous tubules might be similar or were possibly the same cells. Down-regulation of GFRa1 positive cells was obvious in the completely differentiated part of the seminiferous tubule, presenting haploid cells.

We counted the GFRa1 positive cells in the testis sections and determined that about 27% of the testicular tubule germ cells expressed GFRa1. High magnification confocal microscopy analysis showed that GFRa1 was negative in the interstitial tissue cells in the seminiferous tubule of the testis (Fig . 1). SSCs and TSCs were cultivated in distinct media to study *GFRa1* gene expression in these cells. SSCs were isolated from the adult testis after enzymatic digestion and the isolated cells were cultivated in the presence of the above mentioned growth factors. The generated SSCs were characterized according to our previous study (20). The immunocytochemistry (ICC) examination demonstrated that the isolated SSCs were positive for the GFRa1 protein whereas the TSCs were negative (Fig . 2). Quantitative mRNA expression by Fluidigm realtime RT-PCR for the *GFRa1* gene indicated significant expression (P<0.001) of SSCs in comparison to TSCs (Fig . 3A). Similarly, RT-PCR analysis showed that *GFRa1* was clearly expressed in the SSCs, but not in the TSCs (Fig .3B). Flow cytometry analysis results confirmed the expression of GFRa1 on SSCs and demonstrated that about 75% of isolated SSCs expressed GFRa1 (Fig. 4).

**Fig.1:**
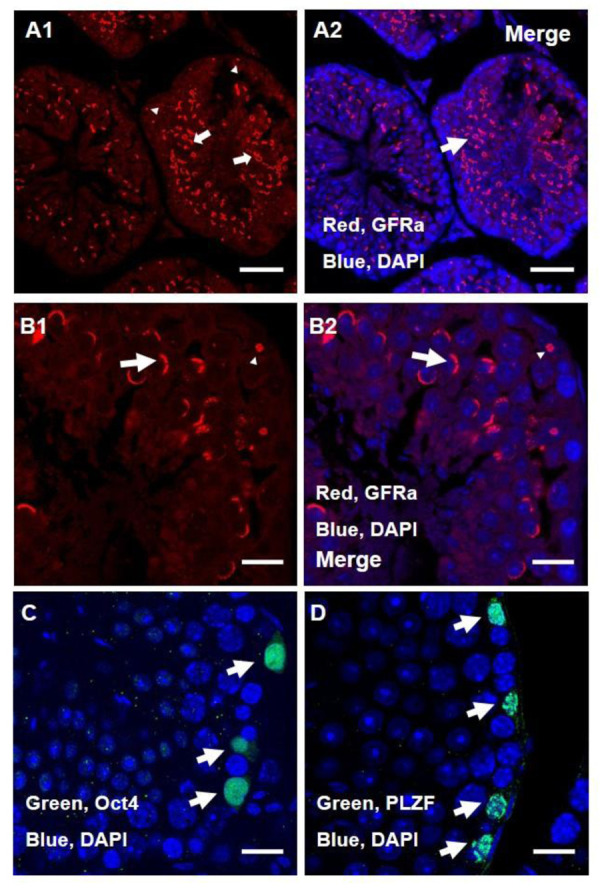
Immunohistochemistry (IHC) analysis of anti-GDNF family receptor alpha 1 antibody (GFRa1) in a testis section. IHC characterization revealed that there are two distinct populations of GFRa1 positive cells. **A1, B1.** The first population expresses GFRa1 with a small round shape in the basal compartment (arrowhead). The second group is located between the basal epithelium and the luminal compartment. This group shows donut and C-shaped expression of GFRa1 (large arrow). Red GFRa1 merges with blue 4', 6-diamidino-2-phenylindole (DAPI), **A2, B2.** Green OCT4 merges with blue DAPI, **C.** PLZF merges with blue DAPI, **D. **According to sections **C and D,** we suggest that PLZF and OCT4 positive cells have similar expression patterns as the first population of GFRa1 positive cells (Scale bar: 50 μm).

**Fig.2:**
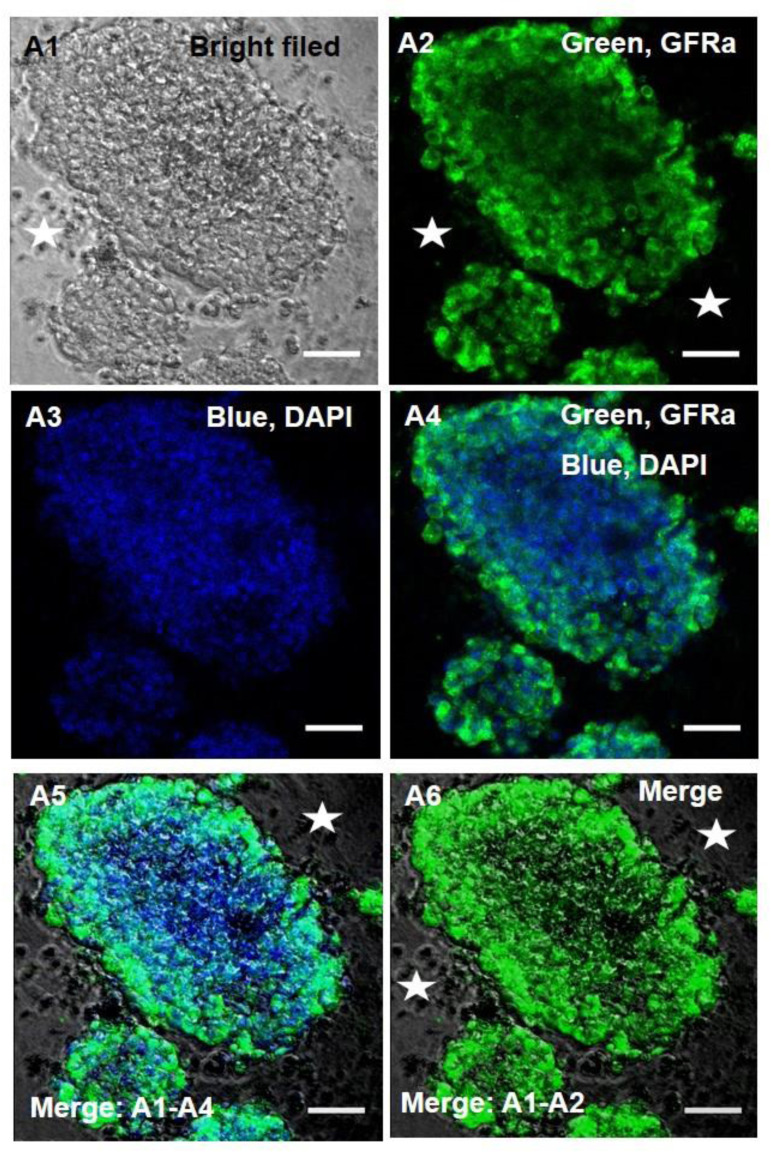
Immunocytochemical analysis of PLZF in spermatogonial stem cells (SSCs) confirmed the expression of anti-GDNF family receptor alpha 1 antibody (GFRa1) in the SSCs and lack of expression in the testicular stromal cells (TSCs) (star). **A1. **Bright field, **A2.** Green fluorescence for PLZF, A3; Blue for 4', 6-diamidino-2-phenylindole (DAPI), and **A4-6.** Merged images (Scale bar: 50 μm).

**Fig.3:**
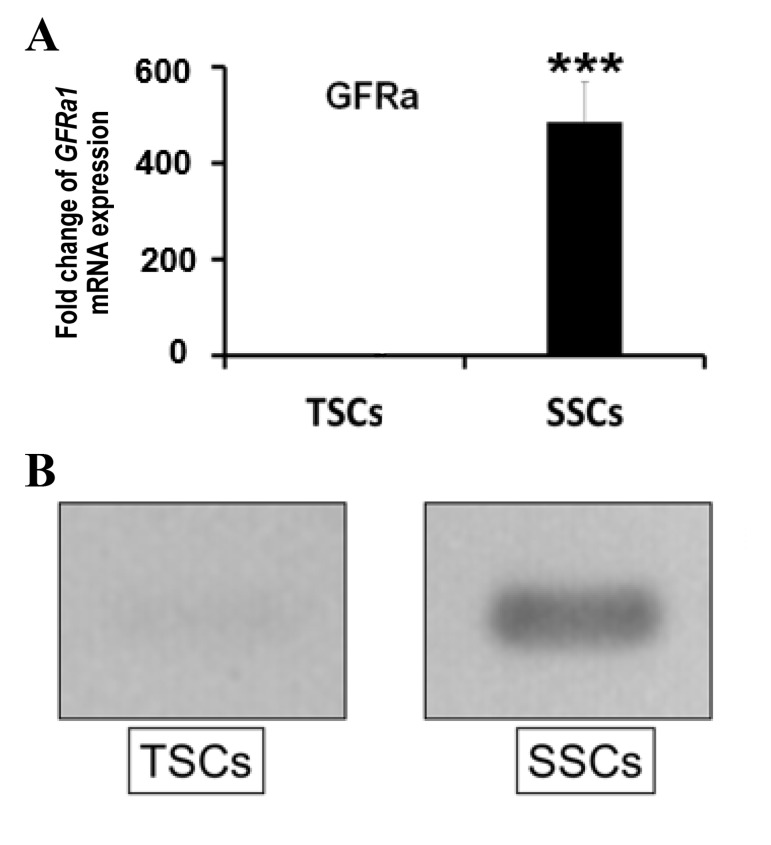
mRNA expression of the anti-GDNF family receptor alpha 1 antibody (*GFRa1*) gene. **A.** Fluidigm real-time PCR (RT-PCR) analysis for *GFRa1* expression in the spermatogonial stem cells (SSCs) and testicular stromal cells (TSCs, P<0.001). Y-axis shows fold change of *GFRa1* mRNA expression in contrast with mouse embryonic fibroblasts. **B. **Reverse transcription polymerase chain reaction (RT-PCR) analysis of *GFRa1* gene for TSCs and SSCs.

**Fig.4:**
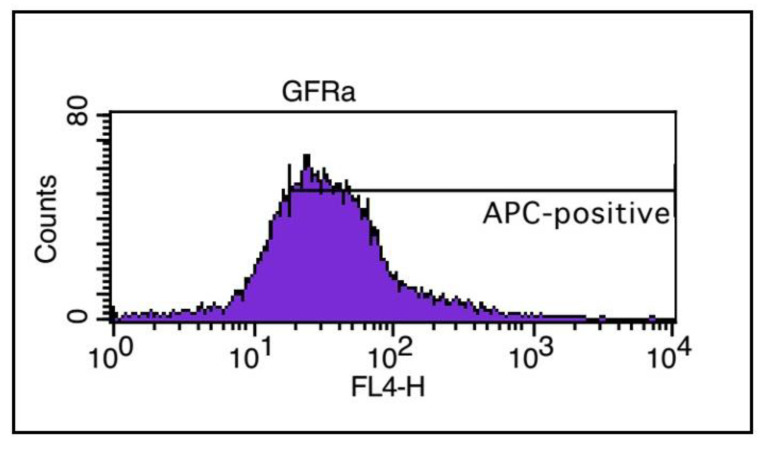
Flow cytometry analysis for anti-GDNF family receptor alpha 1 antibody (GFRa1) expression in the spermatogonial stem cells (SSCs).

## Discussion

Visualization of the testis tissue section by confocal microscopy showed that the germ stem cell marker GFRa1 was localized in these cells above the basement membrane of the testicular lumen. *In vivo* results showed negative expression of GFRa1 in the basement membrane and differentiated part of the testicular lumen. This result showed the heterogeneity of gene expression among undifferentiated spermatogonia during the epithelial cycle (25). A recent study of GDNF regulatory roles on the SSCs fate has shown that GFRa1 and its co-receptor complex, which is located in germ cells, play essential roles during the first wave of spermatogenesis (26). Additionally, as GDNF is involved in SSCs proliferation, it has been suggested that the lack of GDNF or incorrect expression of *GFRa1* would limit colony expansion (27). In a recent study, the results showed elevated GDNF levels when the mitotic activity of undifferentiated spermatogonia was low (28). Kanatsu-Shinohara et al. have suggested that SSCs undergo self-renewal when GDNF is elevated and they undergo differentiation when the GDNF concentration is low (29). Similarly, Sharma and Braun demonstrated that GDNF levels are highest during the stages of SSC proliferation and lowest during the stages of quiescent spermatogonia, which eventually differentiates into A1 spermatogonia (30). While about 27% of testicular tubule cells express GFRa1, negative expression of GFRa1 has been demonstrated in the interstitial tissue cells. Similarly, *in vitro* Assessment revealed that GFRa1 is expressed in SSCs, but not in the TSCs. This finding was confirmed by Fluidigm RT-PCR and ICC. Grisanti et al. reported that 5% of A_paired_ (A_pr_) spermatogonia expressed GFRa1 asymmetrically while 10% of A_single_ (A_s_) did not express GFRa1 (31). As the expression of GFRa1 was obvious between the basement membrane cells and differentiated site of the seminiferous tubules (spermatocytes) of the mouse testes, it seemed that GFRa1 expression was not necessary for the reserved SSCs in the basement membrane and differentiated spermatogonia in the final stage. Therefore, similar to the in vivo model, down-regulation of the GFRa1 germ cell marker might be necessary for the in vitro analysis of SSCs differentiation into sperm. Binding GDNF to the GFRa1 receptor and activating the Ret intracellular signalling pathway regulates the self-renewal and proliferation of SSCs (32). Hasegawa et al. have reported that the stimulation of GFRa1 in the SSCs triggers activation of ERK1/2, which prevents them from differentiation. Similarly, this group demonstrated that the abolished activation of GDNF signalling by the deletion of GFRa1 decreased SSC proliferation (33). It has been proven that GDNF pushes SSC self-renewal by preventing SSC differentiation and not by stimulating proliferation. Activation of GDNF signalling has been shown to increase the phosphorylation of AKT3 in undifferentiated spermatogonia, which led to SSC selfrenewal or progenitor cell expansion (26). By activation of the transcription factors Etv5, Bcl6b and Lhx1 in early spermatogonia, GDNF prevented expression of the c-Kit receptor (34). Production of GDNF and FGF2 by Sertoli cells regulates the self-renewal and proliferation of SSCs, whereas expression of activin A and BMP4 reduces maintenance and promotes differentiation of SSCs (35). During *in vitro* conditions, a different concentration of GDNF (10-100 ng/ml) protein was used for the colony formation of SSCs in culture (35, 36). In the prepubertal testis, interstitial Leydig and peritubular myoid cells express CSF1, which increased the proliferation of undifferentiated SSCs (37).

## Conclusion

Analysis of the data confirmed that the GFRa1 germ cell marker is expressed above the basement membrane of the seminiferous tubule of the testis and in the differentiated section. It seems that GFRa1 is expressed during proliferation and differentiation. According to the roles of GDNF in the regulation of SSCs functions and the potential use for SSCs in the clinical setting, it would be of benefit to conduct future studies on GFRa1 against infertility and other male reproductive dysfunctions. Our results would be helpful for future studies to identify in vitro proliferation and differentiation of SSCs by up- or down-regulation of GFRa1 expression in these SSCs.
